# Integrating CTI and threat modeling for cyber resilience: An AHP assessment

**DOI:** 10.1371/journal.pone.0335154

**Published:** 2025-11-14

**Authors:** Luka Podlesnik, Igor Bernik, Anže Mihelič

**Affiliations:** Faculty of Criminal Justice and Security, University of Maribor, Ljubljana, Slovenia; Oakland University, UNITED STATES OF AMERICA

## Abstract

Cyber resilience has emerged as a critical objective for modern cybersecurity programs, emphasizing not only prevention but also the ability to anticipate, withstand, recover from, and adapt to cyber threats. Two disciplines, cyber threat intelligence (CTI) and threat modeling, are increasingly recognized as fundamental to achieving these goals. While each offers unique benefits, their integration and potential synergistic benefits remain underexplored in empirical research. This study employs the Analytic Hierarchy Process (AHP) to evaluate the comparative effectiveness of an integrated CTI-threat modeling approach versus its standalone implementations. Drawing on judgments from cybersecurity experts across government, finance, and telecommunications sectors, the evaluation is structured around four resilience criteria: anticipate, withstand, recover, and adapt. The results demonstrate a strong preference for integration, particularly in supporting anticipation and operational continuity. These findings provide empirical validation for theoretical arguments and highlight the need for standardized integration frameworks to operationalize this approach and enhance resilience in practice.

## Introduction

Cybersecurity has evolved from focusing primarily on confidentiality, integrity, and availability (CIA) to becoming a strategic capability that is essential for ensuring mission success and organizational continuity [1,2]. This shift is driven by increasingly sophisticated threats such as advanced persistent threats (APTs), supply chain compromises, and geopolitical cyber operations that exploit zero-day vulnerabilities and maintain long-term presence within systems [3–6]. Preventive and control-centric approaches often prove inadequate under these conditions, highlighting the need for cyber resilience.

Cyber resilience reframes the challenge as sustaining essential functions despite disruption by anticipating, withstanding, recovering, and adapting in response to evolving threats. Contemporary resilience approaches reflect this orientation, emphasizing a transition from static defense to adaptive, mission-assured engineering [3,7]. At the core of this reorientation is continuous situation awareness, the capacity to synthesize internal system state and external threat context in real time [8].

Two disciplines are especially relevant to resilience. Cyber threat intelligence (CTI) supports anticipation by analyzing adversary capabilities and intent, but in practice, it is often constrained by ad hoc processes, limited automation, and weak evaluation methods [9,10]. Threat modeling provides a structured way to reason about system vulnerabilities and attack paths, but it frequently remains static and disconnected from real-time intelligence [11,12]. Bidirectional integration may help mitigate these shortcomings, enabling intelligence to dynamically inform models while modeling outputs guide the refinement of intelligence priorities.

Although CTI and threat modeling are each recognized as valuable for advancing resilience, their structured integration has not been developed or tested. Existing studies suggest that complementary benefits could emerge if these disciplines were linked, but provide no formal model or empirical evidence to support this claim [13,14]. Reviews of CTI also emphasize its immaturity as a discipline and the absence of frameworks connecting it to complementary practices such as threat modeling [10]. There is a lack of existing research that directly addresses the structured, bidirectional integration of CTI and threat modeling with respect to measurable resilience outcomes, leaving this as an open and critical area for investigation.

To address these gaps, this article proposes and evaluates a reciprocal integration model in which CTI continuously informs threat models, and these models, in turn, guide intelligence collection. The model is assessed through expert elicitation using the Analytic Hierarchy Process (AHP), structured around the four core goals of cyber resilience: anticipate, withstand, recover, and adapt. This framing leads to the to the following research question: *Does integrating cyber threat intelligence and threat modeling improve cyber resilience more effectively than employing each approach in isolation?* The primary objective of this study is to determine whether the integrated approach yields tangible benefits in resilience outcomes compared to standalone implementations. In doing so, the study aims to establish an initial evidence base for future research, model refinement, and the evaluation of the practical value of CTI-threat modeling integration in advancing cyber resilience.

The remainder of this article is structured as follows: Section Conceptual foundations outlines the principles of cyber resilience and the distinct contributions of CTI and threat modeling. Section Methods details the methodological framework, including the AHP hierarchy and expert elicitation process. The Results section presents the evaluation outcomes. The Discussion section synthesizes the implications, introduces the emergent integration model, and identifies key areas for further research. Finally, the Conclusion summarizes the findings and their significance for advancing cyber resilience practice.

## Conceptual foundations

### Cyber resilience

The concept of cyber resilience has gained increasing attention as the limitations of traditional cybersecurity approaches have become more apparent in the face of complex, dynamic, and evolving threat environments. While cybersecurity has historically focused on maintaining the confidentiality, integrity, and availability (CIA) of information systems through preventive and protective controls, cyber resilience shifts the emphasis toward ensuring mission continuity and adaptive capacity under adverse conditions [1–3,7]. This reframing is particularly relevant when faced with sophisticated cyber attacks and advanced persistent threats (APTs) that can bypass static defenses [1,3].

Where cybersecurity assumes that threats can be effectively identified and neutralized in advance, cyber resilience accepts the inevitability of disruption. Instead, it focuses on the system’s ability to anticipate, withstand, recover from, and adapt to adversity. According to Madni and Jackson, resilience must be viewed not as a static property but as an emergent capability, one that enables a system to function effectively in the face of perturbations and to evolve through experience [15]. Unlike reliability, which emphasizes uninterrupted performance under known conditions, resilience concerns the capacity to reconfigure, re-optimize, or restore operations after disruptions, including those that are unanticipated or novel [3].

Over the past decade, multiple frameworks have sought to define cyber resilience, resulting in a proliferation of overlapping terms across disciplines. A systematic review revealed recurring descriptors such as prepare, resist, respond, recover, and evolve, among others [1]. Table 1 illustrates this diversity by consolidating a representative set of criteria extracted from resilience research. While the table highlights the widespread recognition of resilience as a critical construct, it also makes visible the conceptual fragmentation that hampers empirical assessment. Employing these terms as evaluation dimensions without consolidation risks redundancy and undermines comparability across studies.

**Table 1:** Initial set of cyber resilience-related criteria identified in the literature prior to consolidation.

Criteria	References
anticipate	[3,7,15–21]
prepare	[18,22–24]
plan	[17,23,25]
absorb	[15,17,21–25]
resist	[21,26]
withstand	[3,7,16,18,20,27–29]
continue	[30]
respond	[18,21,26]
recover	[3,7,15–27]
adapt	[3,15–19,21–25,27]
evolve	[7,19,20]

To address this fragmentation, the present study consolidates the terminology into four core criteria: anticipate, withstand, recover, and adapt. Table 2 defines these goals, which align directly with the NIST SP 800-160 and CREF frameworks [3,7]. This consolidation serves two purposes: it reduces redundancy and ambiguity, and it grounds the analysis in standards recognized by both academic and practitioner communities. It also provides the methodological rigor required for the AHP evaluation, enabling structured reasoning about resilience trade-offs while incorporating related concepts (e.g., prepare, continue, transform) as subordinate objectives. As detailed in the Methodology section, this approach mirrors the structure of the Cyber Resiliency Engineering Framework, which organizes eight supporting objectives under the four primary resilience criteria [3,7].

**Table 2:** Consolidated cyber resilience criteria and definitions based on NIST SP 800-160 Volume 2.

Criteria	Definition
Anticipate	Maintain a state of informed preparedness for adversity.
Withstand	Continue essential mission or business functions despite adversity.
Recover	Restore mission or business functions during and after adversity.
Adapt	Modify mission or business functions and/or supporting capabilities in response to predicted changes in the technical, operational, or threat environments.

### Cyber threat intelligence

CTI is defined in this study as the systematic collection, contextualization, analysis, and dissemination of information regarding threat actors, their capabilities, intentions, tactics, and the potential impact of their operations on organizational systems [10,31]. This definition encompasses multiple levels of abstraction, strategic, operational, and tactical, and reflects CTI’s function as a bridge between external threat developments and internal defensive posture.

At the strategic level, CTI supports executive decision-making by illuminating adversary motives, geopolitical trends, and sector-specific threat dynamics [32,33]. At the operational level, it informs threat hunting, incident response planning, and risk management [33,34]. At the tactical level, CTI provides technical indicators such as IP addresses, malware signatures, and indicators of compromise to be used in detection systems and security appliances [34]. These layers, when functioning in concert, serve to enhance both awareness and actionability.

Despite its conceptual promise, CTI remains unevenly implemented in practice. Arikan *et al*. argue that many intelligence workflows lack formal lifecycle structures, leading to inefficiencies in dissemination, validation, and reuse [31]. Mandt likewise highlights the fragmented nature of CTI production and consumption, emphasizing the need for stronger integration with defense operations and system-level priorities [35]. A recent review by Shin and Lowry reinforces this view, identifying inconsistent performance metrics, a lack of automation, and a gap between intelligence production and its use in operational environments as critical deficiencies in current practice [10].

These limitations have direct implications for cyber resilience. CTI is fundamental to the resilience goal of anticipation, by enabling the early detection of adversarial shifts, and to adaptation, by informing system configuration changes and longer-term architectural decisions [3,8,10]. Yet when threat intelligence is disconnected from system modeling or lacks contextual awareness, its value is diminished. As Bellini *et al*. note, effective situational awareness requires not just the availability of intelligence but its integration with the system’s internal state and decision-making mechanisms [8].

To fully support cyber resilience, CTI must function as both a perceptual and a strategic asset. This entails formalized interfaces with modeling processes, continuous feedback to refine collection priorities, and shared taxonomies for describing threat-relevant system behaviors. The extent to which such integration can be achieved is a core focus of this study’s evaluation framework.

### Threat modeling

Threat modeling is a structured process that involves identifying, analyzing, and evaluating potential threats to a system by assessing its architecture, interfaces, and operational environment from the perspective of an adversary. It aims to anticipate how a threat actor might exploit system vulnerabilities to achieve disruptive or damaging outcomes [11,36]. Within this study, threat modeling is conceptualized not just as a compliance or checklist exercise, but as a strategic activity embedded in resilience-focused systems engineering [7].

Traditional threat modeling practices often rely on frameworks such as STRIDE, PASTA, or attack trees to catalog threats and inform mitigation strategies [11,37]. However, from a cyber resilience perspective, the core function of threat modeling expands beyond the enumeration of threats. It involves reasoning about adversarial capabilities, mission-critical interdependencies, and the potential for system degradation, recovery, and transformation [3,7]. This shift reframes threat modeling as a tool for identifying not just where a system might fail, but how it can continue to function and recover under conditions of partial compromise or uncertainty. Bodeau *et al*. argue that threat modeling in the context of cyber resilience must be dynamic, recursive, and context-sensitive, capable of adapting to changes in both the external threat landscape and the internal state of the system. To remain effective, such modeling should integrate real-time intelligence, reflect shifting mission priorities, and account for persistent adversaries, layered defenses, and the need for graceful degradation and reconfiguration under stress [7,16,20].

However, in practice, threat modeling often remains static. Many organizations treat models as artifacts produced early in the life cycle and rarely updated [12,37]. This snapshot approach quickly loses relevance as systems evolve and adversaries adapt. Ross *et al*. and Bodeau *et al*. both argue that such static models are inadequate for resilience, which requires models to evolve in tandem with system and environmental changes [3,16]. Most empirical work in this domain continues to describe methods rather than demonstrating measurable impacts on resilience [11,37].

To fully support cyber resilience, threat modeling must operate as both an anticipatory and diagnostic mechanism. Beyond identifying known attack paths, it should inform strategic decisions about which system components require hardening, which can tolerate disruption, and where architectural redesigns are warranted to enhance system robustness. When integrated with cyber threat intelligence, threat modeling becomes more dynamic, context-aware, and closely aligned with mission assurance objectives.

Synthesizing across these strands, it becomes clear that CTI and threat modeling offer complementary strengths. Their integration has the potential to reinforce and extend their contribution to cyber resilience goals. CTI provides visibility into the external threat environment, while threat modeling structures internal reasoning about vulnerabilities, dependencies, and potential system degradation. When combined, these capabilities could enhance anticipation by linking intelligence with the internal system context, strengthen withstanding by informing defensive priorities, accelerate recovery by guiding response planning with adversary insights, and promote adaptation by feeding lessons learned back into both intelligence requirements and model updates.

Recent work reinforces both the promise and the limitations of current integration efforts. For example, probabilistic attack graphs enriched with CTI feeds demonstrate how adversary intelligence can be incorporated into modeling; however, these approaches remain unidirectional and technical, focusing on a narrow proxy of resilience time-to-compromise rather than broader resilience outcomes [38]. Similarly, efforts to design dynamic CTI architectures for critical infrastructure highlight the potential of adaptive modeling. Still, current implementations are limited to pilot scopes, emphasize dynamic threat or risk scenarios over system-level abstraction for threat assessment, and lack empirical validation of their impact on resilience [39]. Research on explainable artificial intelligence for internet of things security further illustrates the growing interest in resilience-oriented analytics, however CTI and threat modeling are referenced only as overlapping methods rather than as distinct, structurally integrated processes [40]. Together, these studies acknowledge the potential benefits of integration while underscoring that structured, bidirectional approaches and their effects on resilience remain unaddressed.

## Methods

This study evaluates two alternative approaches to improving cyber resilience: one in CTI and threat modeling is dynamically integrated through reciprocal feedback, and another in which they are implemented as standalone, decoupled processes. The evaluation was conducted using the AHP, a structured multi-criteria decision-making methodology well suited to problems involving subjective expert judgment across complex dimensions [41,42].

### Methodological rationale

The decision to use AHP is grounded in both practical and theoretical considerations. First, the integration of CTI and threat modeling is a high-level strategic question that spans technical, organizational, and architectural domains. This makes it poorly suited for single-metric evaluation or simulation-based approaches. Second, collecting large-scale empirical data on such integration remains difficult, as few organizations currently implement mature reciprocal workflows.

Among the various multi-criteria decision-making (MCDM) methods for evaluating cybersecurity strategies, the AHP was selected due to its clarity and effectiveness. IT effectively breaks down complex issues into hierarchical structures and includes validation through the consistency ratio (CR). Unlike other methods that rely on linear aggregation, AHP allows for structured pairwise comparisons, making it particularly useful for small expert panels. This ensures methodological rigor and provides essential interpretability for both academic researchers and practitioners. It has been successfully applied in prior cybersecurity research, including risk assessment [44], smart grid security [45], maritime cyber resilience [46], and information security decision making [47].

### Problem structure: AHP decision hierarchy

**Fig 1:**
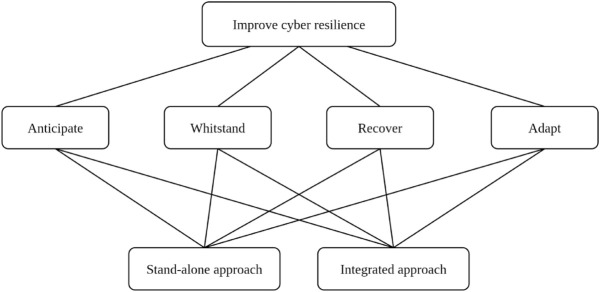
AHP problem structure. Three-level AHP hierarchy to improve cyber resilience, four criteria (Anticipate, Withstand, Recover, Adapt), and two strategic alternatives (integrated vs. standalone).

The decision problem was decomposed into a three-level hierarchy, as illustrated in Fig 1. At the top level, the goal is to improve cyber resilience. The second level defines four evaluation criteria aligned with the NIST SP 800-160 Volume 2 framework: anticipate, withstand, recover, and adapt. The third level contains the two strategic alternatives, integrated and standalone approaches.

### Cyber resilience criteria and objectives

#### Criteria selection and consolidation.

As described in the Conceptual Foundations section, cyber resilience is defined here as the ability of a system to anticipate, withstand, recover from, and adapt to cyber adversity [3,7]. This study adopts these four goals as primary evaluation criteria. The selection was informed by a literature review in which a wide range of resilience-related terms, such as prepare, absorb, resist, respond, evolve, and reconstitute, were identified across academic and practitioner sources (see Table 1).

To reduce conceptual overlap and improve clarity for expert respondents, these terms were consolidated into the four criteria shown in Table 2. This consolidation aligns with the structure of the CREF, which organizes supporting objectives under higher-order resilience functions. By doing so, the evaluation retains conceptual richness while ensuring methodological tractability.

#### Criteria definitions and supporting objectives.

**Fig 2:**
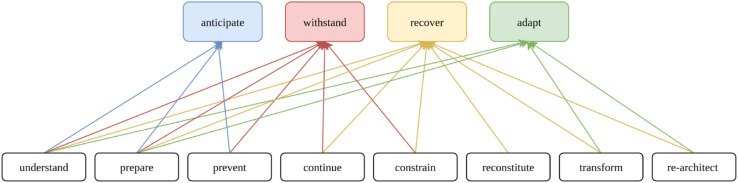
Cyber resilience goals and supporting objectives. Mapping of eight supporting objectives to the four primary cyber resilience goals, illustrating how each goal is operationalized.

While the four resilience goals form the basis for AHP evaluation, they are interpreted through a set of eight supporting objectives: prevent, prepare, continue, constrain, reconstitute, understand, transform, and re-architect [3,7]. These objectives were not treated as independent subcriteria but as interpretive dimensions used to define what each criterion entails in practical terms. Fig 2 illustrates how these objectives align with the four primary goals.

The final criteria definitions used in expert evaluations are listed below:

- **Anticipate**: The capability to identify and prepare for potential threats before they materialize, enabling early action. (Objectives: Prevent, Prepare, Understand)
- **Withstand**: The ability to sustain essential operations despite disruption, minimizing degradation during attack. (Objectives: Continue, Constrain, Prepare, Understand)
- **Recover**: The capacity to restore mission functionality efficiently during or after an incident. (Objectives: Reconstitute, Transform, Constrain, Understand)
- **Adapt**: The ability to modify systems, architectures, or workflows in response to evolving threats or operational changes. (Objectives: Transform, Re-Architect, Understand, Prepare)

### Alternatives

The two strategic alternatives compared in this study are:

#### Integrated approach.

In the integrated configuration, CTI and threat modeling are linked through a bidirectional feedback loop. As described in the Conceptual Foundations section, CTI provides current adversary insights that refine threat models, while model outputs identify gaps and priorities for CTI collection. This approach is designed to support continuous system awareness and proactive adaptation.

#### Standalone approach.

In the standalone configuration, CTI and threat modeling are conducted independently, with no formal integration between them. CTI may be collected based on static intelligence requirements, while threat models may be updated sporadically or not at all. This configuration reflects current practice in many organizations where CTI and security engineering remain operationally disconnected [48].

### Expert elicitation and evaluation process

Expert evaluations were conducted using pairwise comparisons to assess both the relative importance of the four criteria and the relative effectiveness of each alternative under each criterion. To ensure relevant expertise, participants were required to have at least five years of professional experience in CTI, threat modeling, or closely related cybersecurity functions.

Experts were recruited using purposive and snowball sampling via professional outreach. A total of nine responses were collected, of which seven met the predefined experience criteria. The sample size aligns with the established AHP methodology. Saaty and Özdemir argue that for judgment-based evaluations requiring domain expertise, groups of seven to eight participants are often optimal. Larger panels may reduce consistency and dilute expert insight [49,50]. Given the structured nature of AHP and the use of geometric mean aggregation, a seven-member expert panel is both appropriate and methodologically robust.

The aim of this study is not statistical generalization but to provide a structured, proof-of-concept evaluation of CTI-threat modeling integration. Participants were selected from diverse sectors and varied professional backgrounds. The respondents included threat intelligence analysts (n = 4), a CISO or security manager (n = 1), a risk or GRC specialist (n = 1), and a penetration tester (n = 1). The sectoral distribution included government (n = 2), telecommunications (n = 2), finance (n = 1), information technology (n = 1), and cybersecurity and intelligence (n = 1). The levels of experience ranged from 5 to more than 15 years.

Each expert received standardized descriptions of the evaluation criteria and the two strategic alternatives. To minimize cognitive burden and improve consistency, a simplified five-point scale was used for pairwise comparisons, where 1 = equal importance and 5 = extreme importance of one element over the other [51].

Two sets of comparisons were completed:

1. Criterion weighting: Experts compared each pair of resilience criteria to derive their relative importance for improving cyber resilience.
2. Alternative evaluation: For each criterion, experts assessed whether the integrated or standalone approach was more effective.

Individual judgments were aggregated using the geometric mean (Aggregation of Individual Judgments), producing a single reciprocal matrix as shown in Table 3 [52]. The consistency of judgments was assessed using the consistency ratio (CR) as shown in Eq (1), derived from the principal eigenvalue of each matrix. CR values below 0.10 were considered acceptable [42].

$$\text{CR}=\frac{\lambda_{\max}-n}{(n-1)\cdot {\text{RI}}_{n}}$$ (1)

**Table 3:** Consolidated pairwise comparison matrix for cyber-resilience criteria.

	Anticipate	Withstand	Recover	Adapt
**Anticipate**	**1.00**	**1.64**	**0.62**	**3.78**
**Withstand**	**0.61**	**1.00**	**1.79**	**3.16**
**Recover**	**1.62**	**0.56**	**1.00**	**3.12**
**Adapt**	**0.26**	**0.32**	**0.32**	**1.00**

Where $\lambda_{\max}$ is the principal eigenvalue of the matrix, *n* is the number of criteria (*n* = 4), and ${\text{RI}}_{n}$ is the Random Index (RI) for matrix size *n*.

The final global priority scores were calculated using the weighted sum model as shown in Eq (2).

$$P_{i}=\sum_{j=1}^{n}a_{ij}\cdot w_{j}$$ (2)

Where *P*_*i*_ is the global score of alternative *A*_*i*_, *a*_*ij*_ is the local priority of alternative *A*_*i*_ under criterion *C*_*j*_, and *w*_*j*_ is the weight of criterion *C*_*j*_.

All evaluations were conducted using the AHP Online System (AHP-OS), which supports group aggregation, consistency validation, and entropy-based consensus measurement [43]. Group consensus and judgment homogeneity were also measured using Shannon entropy metrics provided by the platform.

### Ethical considerations

This study was reviewed and approved by the Ethics Commission of the Faculty of Criminal Justice and Security, University of Maribor, Slovenia, EU. The ethics approval was issued under reference No.: 1604-2025 on 16 April 2025. The Commission concluded that the research design is ethically sound and compliant with the Code of Ethics and Integrity for Researchers at the University of Maribor. Specifically, the study was found to pose no risk to participants, ensured adequate informed consent procedures, protected personal data, and involved no deception.

Participation in the study was entirely voluntary. All participants received detailed information about the study’s purpose, their rights as participants, and the data confidentiality practices in place. They were informed that they could decline participation or withdraw from the study at any time without consequence. The expert judgments were collected anonymously via an online survey platform between May 5th and July 7th, 2025. No personal or sensitive data were recorded.

## Results

This section presents the outcomes of the expert-based AHP evaluation. The results include: (1) the priority weights assigned to each resilience criterion, (2) the relative performance of the integrated and standalone approaches for each criterion, and (3) the synthesized global priority scores across all criteria. Additionally, the consistency and consensus metrics of the expert judgments are reported to assess reliability.

### Criteria weights and priority ranking

Table 4 shows the relative importance assigned to each of the four cyber resilience criteria. The expert panel assigned the highest priority to anticipate (31.4%), followed closely by withstand (30.7%) and recover (29.4%). The criterion adapt received the lowest weight, at 8.5%. These weights reflect a collective expert judgment that emphasizes early threat identification and operational continuity over long-term system transformation.

**Table 4:** Global priority weights for cyber resilience criteria.

Criterion	Weight (%)	Rank
Anticipate	31.4	1
Withstand	30.7	2
Recover	29.4	3
Adapt	8.5	4

The aggregated pairwise comparison matrix for the criteria yielded a consistency ratio (CR) of 7.9%, which is well below the commonly accepted threshold of 10%, indicating a coherent set of expert judgments. Group consensus on the criterion ranking was measured at 80.5%, suggesting substantial alignment among participants.

### Alternative prioritization by criterion

Table 5 presents the local priority scores of the integrated and standalone approaches under each resilience criterion. Across all four criteria, experts consistently rated the integrated approach as more effective.

**Table 5:** Local priorities of alternatives by criterion.

Criterion	Integrated (%)	Standalone (%)
Anticipate	88.6	11.4
Withstand	86.3	13.7
Recover	67.4	32.6
Adapt	83.8	16.2

The most considerable advantage was observed under the anticipate criterion, where the integrated approach received 88.6% of the weight. This suggests a strong expert belief that real-time threat intelligence, when tightly coupled with dynamic modeling, significantly improves an organization’s ability to foresee and prepare for emerging threats. Similarly, high scores under withstand (86.3%) and adapt (83.8%) indicate that integration enhances both short-term operational robustness and long-term flexibility. Although the margin under recover criterion was narrower, the integrated approach still received a substantial majority (67.4%).

### Overall synthesis and global ranking

The global priority score for each alternative was calculated by combining the local priorities with the corresponding criterion weights using the weighted sum model. As shown in Table 6, the integrated approach received a total score of 81.2%, compared to 18.8% for the standalone approach. This reflects a strong consensus among experts that integrating CTI and threat modeling is more effective for achieving cyber resilience across all evaluated dimensions.

**Table 6:** Final global priority scores of alternatives.

Alternative	Global Priority (%)
Integrated	81.2
Standalone	18.8

### Consensus and consistency assessment

To assess the reliability of expert input, both internal consistency and group consensus were evaluated. The consistency ratio for the criteria matrix was 7.9%, indicating logically coherent judgments. Since comparisons between two alternatives yield automatically consistent matrices, no CR calculation was required for those comparisons.

The group consensus for prioritizing the alternatives was calculated at 87.4%, indicating strong agreement across expert responses. Relative homogeneity of pairwise judgments measured by the standard deviation across responses was 97.5%. These percentages do not represent exact performance metrics but rather standardized measures of consensus and alignment within the AHP process, which are used to evaluate the reliability of expert-based studies [43].

These results reinforce the conclusion that experts perceive the integrated approach as substantially more effective than the standalone approach in improving cyber resilience. At the same time, they should be understood as structured expert judgments rather than definitive measures of system performance.

## Discussion

The central research question guiding this study asked whether integrating cyber threat intelligence and threat modeling improves cyber resilience more effectively than employing each approach in isolation. The findings presented in the Results section affirm this proposition. The integrated approach was strongly favored in expert evaluations, with particular emphasis on its contribution to anticipation and operational continuity. These results provide structured, empirical validation for the conceptual benefits of integration, underscoring its potential to enhance resilience outcomes in both strategic planning and operational execution.

The expert panel assigned the most significant importance to anticipation (31.4%), followed by withstand (30.7%) and recover (29.4%). Adaptation received the lowest weight (8.5%), signaling a shared emphasis on early threat detection and operational continuity over long-term system transformation. This distribution reflects broader patterns observed in resilience engineering literature. Although adaptation is recognized as conceptually vital, it often lacks operational mechanisms, clear ownership, or institutional prioritization [15,30,53,54].

Beyond these structural explanations, organizational and cognitive factors likely contribute to this undervaluation. Experts working in compliance-driven or incident-oriented environments may prioritize visible and immediate outcomes (anticipation, withstanding, recovery) over long-term transformation, which requires cross-functional ownership and delayed return on investment. The results of this study may support this tendency, as the highest scores were assigned to anticipate, withstand, and recover, all of which emphasize near-term visibility and operational continuity. Cognitive framing effects may further bias judgments toward short-term control, while the lack of clear responsibility for adaptation across organizational units makes it less prominent in decision-making. Together, these dynamics suggest that adaptation is not only conceptually underdeveloped but also institutionally underprioritized, underscoring the need for governance structures that elevate adaptation within resilience planning.

### Emergent integration model: Linking CTI and threat modeling

Based on the empirical findings of this study, we propose a conceptual integration model that reflects expert judgments on how CTI and threat modeling should interact to support cyber resilience. Rather than assuming this structure prior to analysis, the model is presented as a synthesized result of the evaluation, which demonstrates a consistent expert preference for integration across all four resilience criteria, particularly for anticipation and operational continuity.

**Fig 3:**
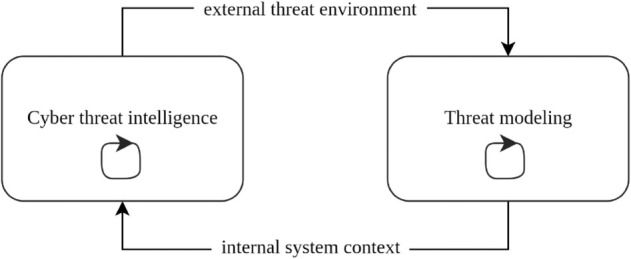
Emergent integration model. A bidirectional feedback loop between CTI and threat modeling enhances analytic monitoring, contextual awareness, and adaptive response.

The proposed model presents an opportunity to overcome the limitations of siloed security practices and enhance cyber resilience through continuous alignment between threat awareness and system posture. The model is structured as a dynamic, bidirectional feedback loop between the CTI and threat modeling processes. As illustrated in Fig 3, CTI provides real- or near-real-time insight into adversary tactics, techniques, and procedures (TTPs), which informs the continuous updating of threat models. These updates may involve revising assumptions, redefining risk scenarios, or reprioritizing architectural defenses.

In turn, threat modeling outcomes, such as newly identified vulnerabilities, critical system interdependencies, or architectural changes, feed back into CTI workflows by refining intelligence requirements and collection priorities. This reciprocal relationship aligns with the resilience mechanisms emphasized in frameworks such as the CREF and NIST SP 800-160 Volume 2 [7], particularly:

- Analytic Monitoring, by ensuring that the threat models reflect current adversarial behaviors and system exposure.
- Contextual Awareness, through the synthesis of external threat data and the state of the internal system.
- Adaptive Response, by enabling timely and evidence-based system reconfiguration or mitigation strategies.

The model also aligns with the principles of situation awareness articulated by Bellini *et al*., who argue that meaningful awareness arises not from isolated data points but from the synthesis of external context and internal state into actionable understanding [8]. CTI, when divorced from system context, risks becoming generic or misaligned; threat modeling, when isolated from current threat data, risks becoming stale or incomplete. Integration, therefore, serves both perceptual and functional goals within resilient security engineering.

Unlike static workflows or unidirectional data flows, this architecture supports a cyber defense posture characterized by learning, responsiveness, and co-evolution with adversaries. The model is not proposed as a finalized solution, but rather as an empirically grounded conceptual architecture to inform further research and operational refinement.

### Implications

This study offers several important implications for both theory and practice at the intersection of cyber resilience, CTI, and threat modeling. By empirically evaluating an integrated approach through structured expert input and AHP analysis, the research advances current understanding of how feedback-driven architectures can enhance cyber resilience capabilities. The findings contribute to theoretical clarification, support the development of methodological tools, and offer actionable guidance for cybersecurity practitioners.

From a theoretical perspective, the study contributes to the validation of long-standing integration hypotheses that have previously lacked empirical testing. While prior work has proposed conceptual models for aligning CTI with threat modeling [7,8,14], these propositions remained speculative. By demonstrating a consistent expert preference for integration across all four resilience goals, particularly for anticipation and operational continuity, this research provides structured support for the proposition that integration can enhance resilience performance, while highlighting the need for further empirical validation.

Second, the study contributes to structuring the empirical evaluation of cyber resilience by applying AHP to the four established resilience goals: anticipate, withstand, recover, and adapt. This approach demonstrates a pragmatic way to assess the relative value of strategic alternatives where empirical system data is scarce. Although AHP relies on expert judgment and should not be treated as a substitute for operational validation, it provides a transparent, replicable framework that can structure comparative resilience assessments in other cybersecurity contexts.

Third, the study helps bridge two historically siloed domains, CTI and threat modeling, within a systems-oriented perspective on cyber resilience. Drawing on principles from resilience engineering and situation awareness theory, the findings support a shift away from static, segmented workflows toward dynamic, feedback-based architectures. This aligns with systems thinking approaches that emphasize the continuous integration of external threat data with internal system modeling to strengthen organizational agility and mission assurance [3,15].

Practically, the results suggest that CTI-threat modeling integration can be treated as a strategic capability investment in environments where early detection and continuity of operations are mission-critical. The strong expert preference for integration under the anticipate and withstand criteria indicates that organizations may gain the most immediate benefit by aligning CTI with modeling to improve foresight and robustness. Over time, integration may also provide a foundation for enhancing adaptation, though this remains underdeveloped and requires organizational commitment to long-term structural change.

Furthermore, the proposed integration model provides a basis for developing adaptive, intelligence-driven, and automation-ready cybersecurity architectures. By linking CTI and threat modeling in a continuous feedback loop, the model supports more agile and responsive defense postures, particularly if augmented by artificial intelligence and machine learning. These technologies could accelerate the mapping of intelligence to models, reduce detection-to-mitigation latency, and improve situational awareness at scale.

Lastly, the findings underscore the importance of organizational governance in maintaining organizational integration. Effective feedback loops between CTI and threat modeling require more than technical interfaces; they require cross-functional collaboration, shared taxonomies, and synchronized update cycles. Institutions aiming to implement integration at scale must establish governance structures that embed this collaboration into routine workflows, bridging the operational divide between intelligence, architecture, and incident response functions.

Collectively, these implications highlight the dual nature of integration, both as a theoretical innovation and as a practical enabler of cyber resilience. The model proposed in this study should be viewed as a foundational step rather than a final solution, inviting further work on its refinement, standardization, and large-scale operationalization.

### Limitations and areas for further research

This study has several limitations that should be acknowledged. First, it relies on expert elicitation using the AHP method, which is inherently sensitive to the selection and composition of the panel. The panel consisted of seven professionals with demonstrated experience across CTI, threat modeling, penetration testing, governance, and related cybersecurity domains. While the sample size is modest, the selection was constrained by the difficulty of identifying individuals with sufficient cross-domain expertise to assess the integrated approach meaningfully. The panel reflects the broader challenge in cyber resilience research, where subject-matter experts are often domain-specialized and rarely possess cross-domain expertise. Nonetheless, the inclusion of both private and public sector experts, combined with strong consensus and low consistency ratios, supports the reliability of the collective judgments.

Second, the AHP method captures structured expert perceptions rather than direct system performance. While this approach is suitable for evaluating conceptual models at an early stage, it implies that the findings should be interpreted as informed judgments rather than empirical performance outcomes. Although the seven-expert group aligns with established AHP practice for domain-specific evaluation [49,50], different panels may produce different results.

Third, integrating CTI and threat modeling also introduces ethical and organizational risks. Intelligence sources may be incomplete, biased, or strategically manipulated, and cognitive or sectoral biases can shape expert judgment. Although this study mitigated such risks through participant diversity and consensus validation, they cannot be eliminated entirely. To mitigate these risks more broadly, organizations should validate intelligence sources, ensure diversity of input, and establish clear governance processes for decision-making.

Finally, the evaluation was explicitly scoped to the four resilience goals: anticipate, withstand, recover, and adapt. While these goals are widely recognized, they do not capture all possible dimensions of resilience. Future studies may therefore consider extending the evaluative framework to include additional resilience capabilities or system-level metrics.

Based on these limitations, several areas for future research can be identified. First, model refinement and technical experimentation are needed to prototype integrated architectures and assess their performance under varying conditions, including diverse threat types, organizational contexts, and mission priorities. This could include testing AI-augmented feedback loops or automating CTI-to-model mappings. Second, research should examine the governance structures and role coordination required for sustainable integration across CTI, threat modeling, development, operations, and risk management functions. This includes exploring workflow orchestration, shared accountability, and secure data exchange under regulatory constraints. Third, longitudinal case studies within organizations are crucial for evaluating the practical impact of integration on resilience outcomes.

By addressing these areas, future research can move beyond conceptual validation and toward a practical roadmap for implementing integration architectures that measurably enhance cyber resilience across sectors.

## Conclusion

This study examined whether integrating CTI and threat modeling improves cyber resilience more effectively than applying each in isolation. Using AHP, expert judgments were elicited and structured to compare two strategic alternatives across the four core resilience goals: anticipate, withstand, recover, and adapt.

The results reveal a strong preference for the integrated approach, most notably in relation to anticipate, withstand, and recover criteria. While integration was favored across all resilience dimensions, these areas emerged as the most prominent. The findings provide structured support for the argument that CTI-threat modeling integration can enhance cyber resilience by strengthening performance across its core objectives.

This study addresses a gap by empirically evaluating CTI-threat modeling integration as a bidirectional mechanism for improving cyber resilience, a largely untested area. In doing so, it contributes to the evolving discourse on resilience-oriented cybersecurity by offering a structured evaluation framework that bridges high-level resilience theory with practical assessment tools.

To transition from conceptual validation to operational impact, further work is required to define how integration can be effectively realized within real-world environments. This includes the development of shared data models, workflow interfaces, and governance structures that support continuous reciprocal feedback. Longitudinal case studies in different sectors will be especially valuable for assessing how integration affects detection speed, recovery effectiveness, and adaptive capacity over time.

Ultimately, the integration of CTI and threat modeling should not be seen as a tooling decision, but as a systems-level capability that enables mission assurance in contested and dynamic threat environments. As cyber threats become more adaptive, persistent, and disruptive, the ability to synthesize intelligence with modeling in a continuous loop may prove essential to maintaining resilience, agility, and trust in complex digital systems.
